# 1247. Molecular Epidemiology of Multi-drug Resistant *Klebsiella pneumoniae* and *K. quasipneumoniae* in Qatar

**DOI:** 10.1093/ofid/ofab466.1439

**Published:** 2021-12-04

**Authors:** Clement Tsui, Fatma Ben Abid, Christi L McElheny, Muna Almaslamani, Abdullatif Al Khal, Ali S Omrani, Yohei Doi, Yohei Doi

**Affiliations:** 1 Weill Cornell Medicine-Qatar, Doha, Ar Rayyan, Qatar; 2 Hamad Medical Corporation, Doha, Ad Dawhah, Qatar; 3 University of Pittsburgh, Pittsburgh, PA; 4 Communicable Disease Center, Doha, Ad Dawhah, Qatar

## Abstract

**Background:**

The molecular epidemiology of carbapenem-resistant *Klebsiella* species is not well investigated in Qatar. The objective of this work was to characterize the genetic context of carbapenemase-producing *Klebsiella* isolates recovered from clinical specimens.

**Methods:**

*Klebsiella* isolates (n=100) were collected at 7 tertiary hospitals from 2015-2017. Identification and susceptibility testing were performed using MALDI-TOF MS and BD Phoenix system, respectively. Whole Genome Sequencing was performed on the Illumina NextSeq platform. Phylogenomic analysis, screening of resistance and virulence genes, and comparison of genetic environment of carbapenemase were carried out.

**Results:**

*Klebsiella pneumoniae* was common (80), followed by *K. quasipneumoniae* (16), *K. aerogenes* (3) and *K. oxytoca* (1). The most prevalent were genes encoding NDM-1 (39), OXA-48 (20), OXA-232 (10) and OXA-181 (12). KPC-2 (3) and KPC-3 (2) were also identified; no carbapenemase-encoding genes could be identified in 15 isolates. Plasmid locations of 24 carbapenemase-encoding genes were determined; *bla*_NDM-1_ was localized on IncFII replicon, while *bla*_OXA-181_ and *bla*_OXA-232_ were commonly associated with ColKP3 plasmids. pOXA-48-like plasmid was detected in 17/20 isolates harboring *bla*_OXA-48_. *bla*_KPC-3_ was located on a contig with ‘traditional’ Tn*4401a* mobile genetic element. Sequence types (STs) were diverse and the ‘traditional’ clonal group (CG) 258 was rare. *K. pneumoniae* ST147 was predominant (13), followed by ST231 (7) and ST11 (5). Nine *K. quasipneumoniae* isolates belonged to ST196 and were highly clonal. The virulence loci such as yersiniabactin (*ybt*) and *rmpA* were not detected within the study’s *K. quasipneumoniae* isolates. Amongst *K. pneumoniae*, there were 50 *ybt*+ isolates; 8 isolates had *rmpA*, and of these, 3 belonged to ST383. *K. pneumoniae* serotype K2, the capsular serotype associated with invasive liver abscess syndrome, was detected in 5 isolates. Genetic relationship of carbapenem-resistant Klebsiella pneumoniae and K. quasipneumoniae isolates in Qatar inferred from core genome SNPs.

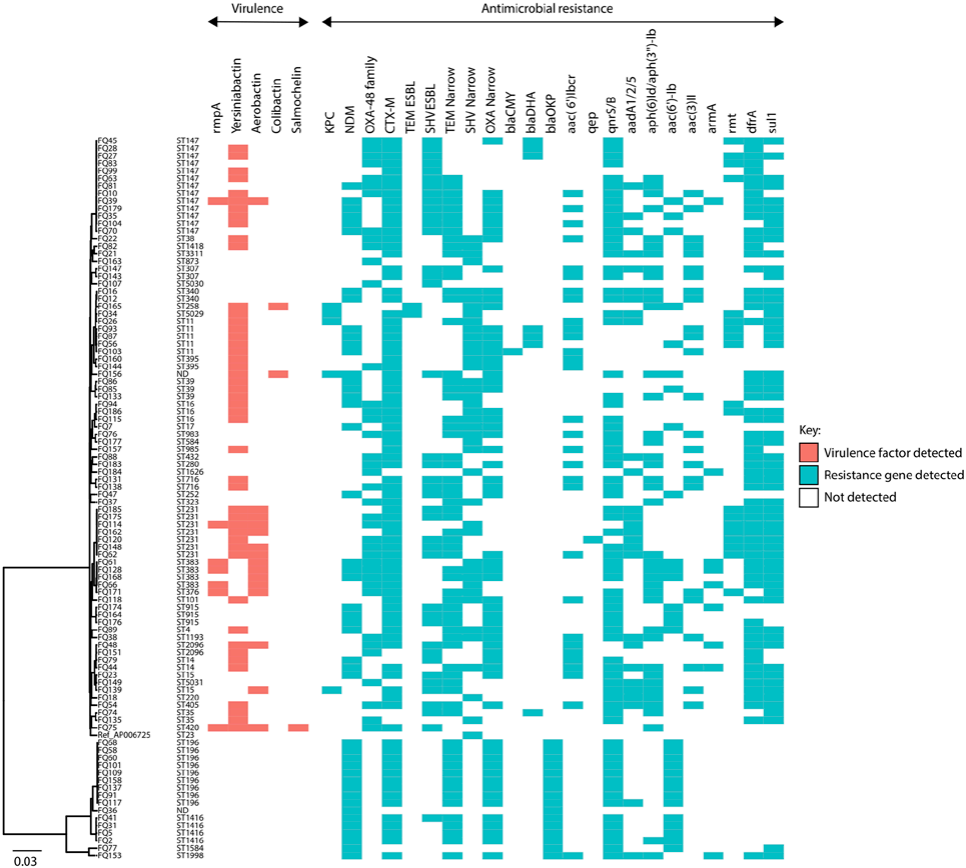

The tree is overlaid with predicted antimicrobial resistance genes and virulence factors for each isolate.

**Conclusion:**

The predominant carbapenemases among clinical *Klebsiella species* isolates in Qatar are NDM and OXA-48 like enzymes, disseminated through various plasmids. The detection of carbapenemase-producing isolate bearing *rmpA* and serotype K2 reflect the presence of both multidrug resistance and hypervirulence in *K. pneumoniae.*

**Disclosures:**

**Yohei Doi, MD, PhD**, **AstraZeneca** (Speaker’s Bureau)**bioMerieux** (Consultant)**FujiFilm** (Advisor or Review Panel member, Speaker’s Bureau)**Gilead** (Consultant)**GSK** (Consultant)**Meiji** (Consultant)**MSD** (Consultant)**Shionogi** (Consultant) **Yohei Doi, MD, PhD**, Astellas (Individual(s) Involved: Self): Grant/Research Support; AstraZeneca (Individual(s) Involved: Self): Speakers’ bureau; bioMerieux (Individual(s) Involved: Self): Consultant, Speakers’ bureau; Chugai (Individual(s) Involved: Self): Consultant; Entasis (Individual(s) Involved: Self): Consultant; FujiFilm (Individual(s) Involved: Self): Advisor or Review Panel member; Gilead (Individual(s) Involved: Self): Consultant; GSK (Individual(s) Involved: Self): Consultant; Kanto Chemical (Individual(s) Involved: Self): Grant/Research Support; MSD (Individual(s) Involved: Self): Speaking Fee; Pfizer (Individual(s) Involved: Self): Grant/Research Support; Shionogi (Individual(s) Involved: Self): Grant/Research Support, Speakers’ bureau; Teijin Healthcare (Individual(s) Involved: Self): Speakers’ bureau; VenatoRx (Individual(s) Involved: Self): Consultant

